# Neurodevelopmental lags and type of delivery in a Colombian infant population

**DOI:** 10.1192/j.eurpsy.2024.1735

**Published:** 2024-08-27

**Authors:** M. Jimenez-Martinez, B. M. Bernal-Gómez, S. Avila-Jimenez

**Affiliations:** 1 Psicologia, Universidad Pedagógica y Tecnológica de Colombia; 2 Psicologia, Grupo Investigación Medición y Evaluación Psicológica en ambientes básicos y aplicados; 3 Medicina, Grupo Investigación Biomedica y de Patologia; 4 Medicina, Universidad Pedagógica y Tecnológica de Colombia; 5 Medicina, Universidad de Boyacá, Tunja, Colombia

## Abstract

**Introduction:**

The Choice giving birth by cesarean section when it is not biologically necessary implies a greater risk to the health of the mother and child Toral *et al. Eletrônica Estácio Saúde 2018; 95*(1) 27-30,refers the psychological relevance to identify perinatal effects of a good medical practice at birth. In this respect Poojari *et al.* Early Hum Dev 2019;115 93-98, state that a cesarean section as a surgical risk, causes decrease fetal oxygenation and an impairment release of stress-related hormones in the maternal-fetal binomial that does not favor neural connections at birth

**Objectives:**

Identify the neurodevelopmental lags in infant on children under 24 months of age born by cesarean section and vaginal delivery,

**Methods:**

A cross-sectional descriptive correlational; Sample consisted of 100 children of a term gestation, 70 with spontaneous vaginal birth and 30 whose birth was by cesarean section, aged between one and twenty-four months; using the Abbreviated Development Scale, an instrument created and validated for the Colombian population (Cronbach’s alpha, 0.94). All parents signed the informed consent.

**Results:**

All test scales were applied (gross and fine motor, language and social personal), the results showed that children born by cesarean section had better development in areas of fine motor and language, while children born by vaginal delivery had better development of gross motor. See (graphic 1).

*Graphic 1: Areas of development according to the type of delivery.*

**References**

**Conclusions:**

The influence of contextual variables such as age and educational level of the mother on language and social areas was also found

**Disclosure of Interest:**

None Declared

